# Mass radical treatment of a group of foreign workers to mitigate the risk of re-establishment of malaria in Sri Lanka

**DOI:** 10.1186/s12936-020-03419-x

**Published:** 2020-09-25

**Authors:** Manonath M. Marasinghe, Vissundara M. Karunasena, Arundika S. Seneratne, Hema D. B. Herath, Deepika Fernando, Rajitha Wickremasinghe, Kamini N. Mendis, Dewanee Ranaweera

**Affiliations:** 1 Anti Malaria Campaign, Colombo, Sri Lanka; 2 Regional Malaria Office, Moneragala, Sri Lanka; 3 Department of Parasitology, Faculty of Medicine, University of Colombo, Colombo, Sri Lanka; 4 Department of Public Health, Faculty of Medicine, University of Kelaniya, Colombo, Sri Lanka; 5 141, Jawatta Road, Colombo 5, Sri Lanka

**Keywords:** Prevention of reintroduction, Malaria, G6PD, Mass radical treatment, Migrant labour

## Abstract

**Background:**

Following malaria elimination, Sri Lanka was free from indigenous transmission for six consecutive years, until the first introduced case was reported in December 2018. The source of transmission (index case) was a member of a group of 32 migrant workers from India and the location of transmission was their residence reporting a high prevalence of the primary vector for malaria. Despite extensive vector control the situation was highly susceptible to onward transmission if another of the group developed malaria. Therefore, Mass Radical Treatment (MRT) of the group of workers for *Plasmodium vivax* malaria was undertaken to mitigate this risk.

**Method:**

The workers were screened for malaria by microscopy and RDT, their haemoglobin level assessed, and tested for Glucose 6 phosphate dehydrogenase deficiency (G6PD) using the Care Start RDT and Brewers test prior to treatment with chloroquine (CQ) 25 mg/kg body weight (over three days) and primaquine (PQ) (0.25 mg/kg/day bodyweight for 14 days) following informed consent. All were monitored for adverse events.

**Results:**

None of the foreign workers were parasitaemic at baseline screening and their haemoglobin levels ranged from 9.7–14.7 g/dl. All 31 individuals (excluding the index case treated previously) were treated with the recommended dose of CQ. The G6PD test results were inconclusive in 45% of the RDT results and were discrepant between the two tests in 31% of the remaining test events. Seven workers who tested G6PD deficient in either test were excluded from PQ and the rest, 24 workers, received PQ. No serious adverse events occurred.

**Conclusions:**

Mass treatment may be an option in prevention of reintroduction settings for groups of migrants who are likely to be carrying latent malaria infections, and resident in areas of high receptivity. However, in the case of *Plasmodium vivax* and *Plasmodium ovale*, a more reliable and affordable point-of-care test for G6PD activity would be required. Most countries which are eliminating malaria now are in the tropical zone and face considerable and similar risks of malaria re-introduction due to massive labour migration between them and neighbouring countries. Regional elimination of malaria should be the focus of global strategy if malaria elimination from countries is to be worthwhile and sustainable.

## Background

A significant feature in the prevention of reintroduction (POR) phase of malaria in Sri Lanka is the high receptivity and vulnerability in parts of the country owing to the persistence of malaria vectors and the high rate of importation of malaria cases [1]. Even though an effective surveillance system is in operation, these conditions impose a significant risk of malaria re-introduction post-elimination [2]. Following the elimination of malaria in 2012 and being free from indigenous transmission for six consecutive years, Sri Lanka reported the first case of introduced malaria in December 2018 [3], the source of the infection (index case) being one of a group of foreign workers from India who developed a *Plasmodium vivax* infection a few days after arriving in Sri Lanka. The location of transmission to the introduced case was a factory construction site in the District of Monaragala, a highly receptive remote area in southwestern Sri Lanka which was previously endemic for malaria; here a group of 32 workers of Indian origin including the index case were employed and resident. The area was found to have a high prevalence of *Anopheles culicifacies*, the primary vector of malaria in the country [3]. Even though extensive measures including vector control and vigilance on malaria cases were introduced in the area following the detection of the introduced case, the situation was highly susceptible to onward transmission if another of the group of individuals developed malaria in the period that followed. Therefore, Mass Radical Treatment (MRT) of the entire group of workers for *Plasmodium vivax* malaria was considered as a measure to mitigate this risk. The objective of MRT was to eliminate any prevailing erythrocytic, sexual or dormant hepatic stages of *P. vivax* in the group of foreign individuals in the factory, thereby reducing the risk of blood infections occurring by way of relapses or recrudescences and initiating a cycle of transmission in the area.

## Methods

### Mass radical treatment for *Plasmodium vivax*

The 32 foreign workers in the factory were formerly residents of Uttar Pradesh, India. One of them was an engineer and the rest were skilled manual workers of various categories. The option of radical treatment was presented to the group of foreign workers in a discussion with them, explaining the probability of them being infected. The reasons for treatment was explained as being both preventing them developing malaria at any point in time in the future and also reducing the risk of onward transmission in the event of a blood infection. All 31 of them consented to undergo treatment. The decision to treat the group radically for *P. vivax* was then taken on the basis of wide consultation and a recommendation of an expert committee, the Technical Support Group of the Anti Malaria Campaign (AMC) [4]. Permission to carry out the mass radical treatment was obtained from the Director General Health Services and Regional Director of Health Services of the area, and agreement was reached on this intervention with the regional health administration and factory management and the workers themselves.

The treatment options chosen were chloroquine (CQ) (600 mg base, 600 mg base and 300 mg base for three consecutive days) and primaquine (PQ) 0.25 mg/kg body weight per day for 14 days after testing for Glucose 6 phosphate dehydrogenase deficiency (G6PD) status in accordance with the national treatment guidelines for *P. vivax* [5]. The choice of CQ was based on the fact that *P. vivax* accounts for a majority of malaria infections in India and that it is still sensitive to CQ [6–8].

By way of preparation for the MRT, the local hospital in the vicinity of the factory and the General Hospital in the District were made aware of the mass radical treatment programme. Discussions were held with the Consultant Physicians and the Haematologists of the catchment hospitals in order to enable preparatory measures to be taken to manage any patient who may develop acute haemolysis following PQ therapy. The medical officers and nursing staff of the local hospital were given a refresher training in identifying haemolysis and arrangements were made to immediately transfer any patient with suspected haemolysis to the nearest tertiary care centre with more facilities for further management. Twenty-four hour transfusion facilities were ensured during the time period of MRT and the Consultant Hematologist of the General Hospital was notified on the possibility of reporting a patient with haemolytic anaemia during the given time period. An awareness programme was carried out for the workers and the factory management regarding the mass radical treatment and the side effects that may be expected. The AMC staff was contactable by telephone at any time in case of an emergency. Information sheets describing the need for MRT, the medicines that will be given and adverse effects that could be expected were prepared along with consent forms. Data were recorded daily in a predesigned data collection form manually as well as electronically. Inclusion criteria for MRT were: (a) all migrant workers currently working in the factory (b) who gave consent for treatment; and (c) had no identifiable contraindications. The contraindications were defined as.

i. Persons with known allergies to medicines used for MRT (CQ and PQ)
ii. Severely ill persons
iii. Persons treated with the same medication regimen after they arrived in Sri Lanka (with documented evidence)
iv. History suggestive of haemolysis after taking anti-malarial medication

Informed written consent was obtained from all the foreign workers prior to treatment. On the basis of these criteria all 31 persons were found to be eligible for treatment excluding the index case who was treated radically during the malaria infection.

Fourteen days of PQ warranted prior exclusion of moderate to severe G6PD deficiency [5]. The reported prevalence of G6PD deficiency in a selected population of a District of Uttar Pradesh was 13% [9]. All eligible persons were subjected to two tests for G6PD activity—G6PD Rapid Diagnostic Test (RDT) (Care start ®) and the Brewers test, and those who were positive on either or both of the tests were excluded from PQ treatment. Both CQ and PQ were administered as directly observed treatment (DOTs).

All individuals were tested for malaria by microscopy using Giemsa-stained thick and thin blood smears and Pan-specific RDT (Care start™) prior to commencement of treatment. A baseline haemoglobin level was performed by a laboratory auto-analyzer before commencement of treatment.

## Results

All 31 eligible foreign workers in the factory gave informed consent for radical treatment. None of them were parasitaemic at baseline screening. Their haemoglobin levels at baseline ranged from 9.7 to 14.7 g/dl (median 13.7; and mean 13.3 g/dl). All 31 individuals were treated with the recommended daily regime of CQ for three consecutive days.

The results of the G6PD screening are given in Table 1. Fifteen test events were inconclusive in one or the other test, and when these were excluded the results were discrepant between the two tests in 5 of the 16 (31%) persons. Seven workers, who were found to be G6PD deficient by one or the other or both of the tests performed, were excluded from PQ treatment. The rest, 24 workers were given the full course of PQ at 0.25 mg/kg body weight for 14 days. Treatment with PQ was commenced after an interval of one day after the CQ regimen was completed in order to allow for recovery from minor adverse reactions to CQ if any. Subjects were monitored daily for the occurrence of adverse effects including symptoms of haemolysis until completion of the treatment and all were followed up at monthly intervals by microscopy for a period of 5 months.

**Table 1:** Results of G6PD testing by method

Test	G6PD enzyme not deficient (%)	G6PD enzyme deficient (%)	Inconclusive (%)	Total
Brewer’s test	29 (93.5)	1 (3.2)	1 (3.2)	31
G6PD RDT	11 (35.5)	6 (19.3)	14 (45.1)	31

No major adverse effects were reported during or after the treatment. Mild adverse reactions were reported during treatment with CQ as given in Table 2, but none were reported during the period of PQ treatment.

**Table 2:** Minor adverse effects reported during treatment with chloroquine

Symptom	Number of people reporting an effect during the period of CQ treatment (%)
Headache	8 (25.8)
Abdominal pain	4 (12.9)
Nausea	4 (12.9)
Dizziness	2 (6.5)
Weakness	1 (3.2)
Feeling faint	1 (3.2)
Joint pains	1 (3.2)
Lose or semi-formed stools	1 (3.2)
Breathlessness	1 (3.2)
Other	2 (6.5)

All 31 individuals, treated (n = 24) and untreated (n = 7) with PQ remained aparasitaemic during the next 5 months of follow up.

## Discussion

This study reports on the successful implementation of an intervention comprising mass radical treatment for *P. vivax* of a group of 31 individuals from India, resident and working at a factory construction site in a previously malarious area of Sri Lanka. The purpose of this intervention was to mitigate the risk of malaria re-establishment in Sri Lanka. The risk was proven by the occurrence of a *P. vivax* infection among one member of this group of foreign workers (an index case), and onward transmission from that infection to a Sri Lankan national leading to the first case of introduced malaria in Sri Lanka 6 years after malaria elimination [3]. The intervention of MRT was warranted on the grounds that (1) there remained 31 other members of the group of foreign workers who originated from the same region of India as the index case who, therefore, may have also harboured latent hepatic stage malaria parasites or even sub-clinical and sub-patient blood infections. These could manifest as clinically and parasitologically patent infections at a later time, as did the index case. (2) This coupled with the fact that the area where they were resident had a high prevalence of the primary vector of malaria in Sri Lanka, constituted a risk for resumption of transmission. Even the extremely thorough surveillance and case management system for malaria in place in the country at present [1, 10] and the vector control measures that were implemented at the factory site may not have been sufficient to mitigate the risk of reintroduction because of rampant population movement in the country, as testified by the occurrence of the introduced case.

Apart from alleviating the risk of transmission in the host country, ethical grounds for mass treatment of such groups of migrant labour or refugees, are believed to be substantial, given the benefits that would accrue to the treated individuals. If there is evidence that a group of individuals originates from a malaria endemic region of a country, and thus are at a moderate to high risk of developing a malaria blood infection while they are in the recipient country, the individual benefits of mass treatment of the group are likely to exceed the risks of treatment, for the following reasons: firstly, the stress induced by travel to the country of work, and the often arduous work they have to be engaged in thereafter, increases, considerably, the chances of developing a clinical malaria infection from dormant parasite stages, on arrival in the country. This is supported by the fact that several of the imported malaria cases in Sri Lanka in the past few years have developed malaria within a few days or weeks of their arrival in the country, although they have been malaria-free for many months and even a year prior to arrival. Secondly, if they do fall ill, being foreign citizens, they may not have the same degree of access to diagnosis and treatment facilities in the recipient country as do the nationals. Unless the employing company or agency provides them with a sound health care cover, which is often not the case, they may in general not be able to have ready access to malaria diagnosis and treatment. Sri Lanka recently adopted a policy of providing diagnosis and treatment for malaria as in-patients to foreign persons free-of-charge at any government health institution, making an exception to the rule that foreigners are not otherwise eligible for free health care in the government sector. Furthermore, the recently adopted National Migration Health Policy of Sri Lanka [11] allows for the provision of a defined package of health services to legally imported labour, but illegal migrant labour groups which are many, are not covered by this policy. Therefore, mass treatment of migrant labour or refugee groups may be justifiable on the grounds that the benefits to the individual are likely to exceed the risks, provided that the chances of them being infected with malaria can be established reasonably well. A history of residence in a malaria endemic country or region, may not, by itself, be a sufficient indication that individuals in a group are likely to harbor a latent malaria infection. The Anti Malaria Campaign is, therefore, currently working on validating a serological assay that could serve as a more reliable marker of the degree of exposure of a group of individuals to malaria prior to arrival.

Although in the particular situation described here, MRT was executed and was well tolerated by the individuals with no occurrence of serious adverse events, the procedure faced many operational and technical challenges. Tools to screen for G6PD activity prior to treatment with PQ, the only registered anti-relapse medicine today, were not optimal. Not only were the results inconclusive in a high proportion of the RDTs performed, nearly a third of the results were discrepant between the two tests. These were the only tests that were available and feasible to use in this situation, and neither were quantitative. Therefore, using the approach of an abundance of caution, almost a quarter of the group of individuals had to be excluded from PQ treatment on the grounds that they tested positive for G6PD deficiency on either or both tests. Until a more reliable, quantitative and affordable point-of-care test is available for use in such situations, the strategy of mass treatment of groups at high risk of developing *P. vivax* malaria may not be feasible in most POR settings.

Preparations for the mass treatment undertaken here were extensive. They included comprehensive planning, communication with the individuals and their employer on the purpose, benefits and risks of treatment, and taking elaborate measures in a remote area of the country to detect and deal with any life-threatening adverse events by way of haemolysis that could have occurred as a result of PQ treatment, despite screening for G6PD activity, albeit with less-than-optimal qualitative tests. All these procedures were costly in terms of time, effort and commodities. It is difficult to imagine that such an intervention can be undertaken on a scale, wide enough to mitigate the risk imposed by the large numbers of migrant labour groups present in the country.

Surveillance, both parasitological and entomological is one of the key strategies used by the AMC to maintain zero transmission since the elimination of malaria in 2012 [1]. With 378 imported malaria cases being diagnosed between 2013 and 2019, the country remains vulnerable to the re-introduction of malaria. The AMC has thus far been successful in detecting high-risk groups, which include foreign labour, asylum seekers from South Asia, fishermen from Sierra Leone [12], security forces personnel returning from UN Peacekeeping missions in malaria endemic countries [13, 14] and war-displaced Sri Lankan returnees from India and providing treatment to those who are positive. The AMC and Regional Malaria Officers are continuously engaged in identifying new groups of individuals who could re-introduce malaria to Sri Lanka and screening them periodically for malaria. Mass screening and treatment is a costly and labour intensive procedure and not an entirely effective one either, because latent malaria infections are not detected by blood screening, and could, as experience has shown, manifest at a later time. Passive case detection of malaria has also been strengthened in the country, yet it faces major challenges such as delays in diagnosis, owing to malaria being a rare disease in the country [2, 15, 16].

The role of Mass Drug Administration (MDA) in malaria elimination has been given thorough consideration, with policy recommendations from the World Health Organization [17] and also subjected to extensive debate [18, 19]. A distinction has to be drawn between MDA for malaria elimination and the intervention of mass radical treatment reported here, in that it was used here as a means of preventing the resumption of transmission in an already malaria-free setting and not as a tool for elimination. MRT was undertaken here to address a situation which increasingly faces countries in the tropical belt particularly, but not confined to Asia, which are now eliminating malaria. This is that with massive developmental projects underway in most countries, there is rampant human movement, largely by way of migrant labour between these countries. Those countries in Asia that are now eliminating malaria, such as Timor Leste, Bhutan and Nepal, are in the tropical belt and they remain highly vulnerable to the re-establishment of malaria due to the presence of malaria vectors, and groups of migrant labour who move freely between them and their neighbouring countries (Indonesia and India respectively) with ongoing transmission [20–22]. They often reside in the recipient country for several months offering a continuous source of parasites. It has been the experience in Sri Lanka during the past six years since elimination, that many imported malaria patients in such groups and even international travellers and refugees, who have often been screened for malaria at various points in time after arrival in the country, have reported negative on screening but developed malaria infection and illness subsequently. This is to be expected given that, particularly in the case of *P. vivax* and *Plasmodium ovale*, dormant hepatic stages which can later lead to blood infections cannot be detected by screening. More proactive measures to mitigate the risk of re-establishment of malaria from migrant labour and refugee groups beyond active and reactive screening are very much needed and may not be optional strategies in countries which are now eliminating malaria. Although they are expensive and labour-intensive such measures would still cost the country far less than if malaria were to return. Hindsight of Sri Lanka’s experience in the 1960s [23], when the disease resurged after near elimination, and persisted for 50 years costing the country dearly in terms of health and human development is testimony to this argument. Mitigating the risk of re-establishment of transmission is a serious problem in countries in the tropics that eliminate malaria, at least until entire regions become free of malaria [2]. This calls for greater efforts to eliminate malaria from regions in order to make country elimination efforts sustainable and worthwhile.

## Conclusions

Mass treatment is an option for use in POR settings for large groups of migrants who are likely to be carrying latent malaria infections, and are resident in areas where the receptivity is high. However, when this applies to *P. vivax* and *P. ovale* a more reliable, quantitative and affordable test to detect G6PD activity at the point-of-care will be needed to ensure that the risk–benefit ratio of this intervention is sufficiently low to warrant its use.

## Data Availability

The data and material are available with the Director of the Anti Malaria Campaign.

## Abbreviations

AMC: Anti Malaria Campaign
CQ: Chloroquine
DOTS: Directly Observed Treatment Strategy
G6PD: Glucose 6 Phosphate Dehydrogenase Deficiency
MDA: Mass Drug Administration
MRT: Mass Radical Treatment
POR: Prevention of reintroduction
PQ: Primaquine
RDT: Rapid Diagnostic Tests
